# Polysaccharide-Containing Macromolecules in a Kampo (Traditional Japanese Herbal) Medicine, Hochuekkito: Dual Active Ingredients for Modulation of Immune Functions on Intestinal Peyer's Patches and Epithelial cells

**DOI:** 10.1093/ecam/nep193

**Published:** 2011-03-10

**Authors:** Hiroaki Kiyohara, Kazuki Nonaka, Michiko Sekiya, Tsukasa Matsumoto, Takayuki Nagai, Yoshiaki Tabuchi, Haruki Yamada

**Affiliations:** ^1^Kitasato Institute for Life Sciences & Graduate School of Infection Control Sciences, Kitasato University, Minato-ku, Tokyo 108-8641, Japan; ^2^Oriental Medicine Research Center, Kitasato University, Tokyo 108-8641, Japan; ^3^Faculty of Pharmacy, Iwaki Meisei University, Fukushima 970-8551, Japan; ^4^Life Science Research Center, University of Toyama, Toyama 930-0194, Japan

## Abstract

A traditional Japanese herbal (Kampo) medicine, Hochuekkito (Bu-Zhong-Yi-Qi-Tang in Chinese, TJ-41) is a well-known Kampo formula, and has been found to enhance antigen-specific antibody response in not only local mucosal immune system in upper respiratory tract, but also systemic immune system through upper respiratory mucosal immune system. Although this immunopharmacological effect has been proposed to express by modulation of intestinal immune system including Peyer's patches and intestinal epithelial cells, active ingredients are not known. TJ-41 directly affected the production of bone marrow cell-proliferative growth factors from murine Peyer's patch immunocompetent cells *in vitro*. Among low molecular, intermediate size and macromolecular weight fractions prepared from TJ-41, only fraction containing macromolecular weight ingredients showed Peyer's patch-mediated bone marrow cell-proliferation enhancing activity. Anion-exchange chromatography and gel filtration gave 17 subfractions comprising polysaccharides and lignins from the macromolecular weight fraction of TJ-41, and some of the subfractions showed significant enhancing activities having different degrees. Some of the subfractions also expressed stimulating activity on G-CSF-production from colonic epithelial cells, and statistically significant positive correlation was observed among enhancing activities of the subfractions against Peyer's patch immunocompetent cells and epithelial cells. Among the fractions from TJ-41 oral administration of macromolecular weight ingredient fraction to mice succeeded to enhance antigen-specific antibody response in systemic immune system through upper respiratory mucosal immune system, but all the separated fractions failed to enhance the *in vivo* antibody response in upper respiratory tract.

## 1. Introduction

A formula of traditional Japanese herbal (Kampo) medicine, Hochuekkito (Bu-Zong-Yi-Qi-Tang in Chinese, TJ-41) comprises 10 kinds of single herbs, and has been used traditionally for the treatment of weak patients, who have chronic diseases possessing symptoms such as loss of appetite, mild fever, night sweat, palpitation, fear, restlessness, weak feeble voice, slurred speech and disturbance of vision [1]. The medicine is intended to be applied for patients bearing the symptoms in respiratory apparatus [2], and given clinical efficacies for treatments of chronic cold, prevention of respiratory infection with MRSA on patient having impaired consciousness [3], prevention of respiratory infection after surgery [4], and improvement of chronic obstructive pulmonary disease (COPD) [5, 6] on modern medical care in Japan.

In upper respiratory tract, specified local mucosal immune system exists, and nasal immunization with antigen induces antigen-specific plasmablasts in nasal-associated lymphoreticular tissue (NALT) [7]. The plasmablasts migrate into systemic blood stream through jugular lymphatic trunk, and are mainly distributed into spleen and peripheral lymph nodes as systemic immune system in addition to lamina propria of upper respiratory mucosal immune system due to interaction between several homing receptors and their ligands for lymphocytes [7–9]. Finally, antigen-specific antibody forming (plasma) cells are produced in these local immune systems [7, 8]. Recently, we have found that TJ-41 enhances not only antigen-specific secretory IgA antibody production in upper respiratory mucosal immune system, but also antigen-specific immunoglobulin (IgG) production in systemic immune system against nasal immunization with antigen [10]. This immunopharmacological effect of TJ-41 was abrogated by impairment of intestinal immune system by oral administration of methotrexate [10]. Intestinal mucosal immune system includes Peyer's patches and isolated lymphoid follicles (ILF) as the specialized secondary lymphoid organs [7]. These lymphoid organs play as inductive sites for mucosal immune system, and B and T lymphocytes migrate from the organs through mesenteric lymph node and thoracic duct to the other local mucosal immune system including upper respiratory mucosal immune system and systemic immune system [7, 11]. Matsumoto et al. [12] have found that TJ-41 also enhances antigen-specific antibody response against oral immunization in not only intestine but also serum and nasal cavity. It also has been clarified that proportion of CD62L-positive B lymphocytes in Peyer's patches and blood stream significantly increased by oral administration of TJ-41 to mice [12]. These facts postulates that active ingredient(s) in TJ-41 interact with immunocompetent cells in Peyer's patches to modulate homing receptor expression and/or cytokine production, resulting in the stimulation of upper respiratory mucosal immune system through certain bystander effects of lymphocytes that are recruited from intestinal immune system. Therefore, it is believed that Peyer's patches are one of first target organs for expression of stimulating activity of TJ-41 on upper respiratory and intestinal mucosal immune systems.

Meanwhile, intestinal lumen are covered by numerous epithelial cells, and the cells also have been clarified to play roles as inductive site of mucosal immune system through expressions of MHC class I and II, some adhesive molecules like VCAM-1, and several cytokines to crosstalk with underlying lymphocytes and dendritic cells in lamina propria [13, 14]. Dendritic cells in lamina propria migrate into mesenteric lymph nodes, and the resulting cells interact with both lymphocytes and dendritic cells that are recruited from Peyer's patches, then lymphocytes in mesenteric lymph nodes are circulated to populate mucosal and systemic immune systems through thoracic duct [11, 15]. TJ-41 has been shown to enhance G-CSF production of both murine normal colonic epithelial cell-line (MCE301) and primary-cultured murine colonic epithelial cells [16]. Therefore, it is hypothesized that active ingredients in TJ-41 interact with both epithelial cells and immunocompetent cells in Peyer's patches, and resulting to potentiate distal mucosal and systemic immune systems by migration of functionally modulated lymphocytes through mesenteric lymph nodes from Peyer's patches. Although it has been proposed that macromolecular weight ingredients may contribute to expression of G-CSF production enhancing activity against epithelial cells [16], the active ingredients in TJ-41 for modulation of Peyer's patch immunocompetent cells are not known.

This article deals with clarification of active ingredients in TJ-41 for modulation of immunocompetent cells in Peyer's patches, and polysaccahride-containing macromolecular ingredients in TJ-41 were found to be dual active ingredients for Peyer's patch cells and intestinal epithelial cells.

## 2. Methods

### 2.1. Materials

**Figure 1:**
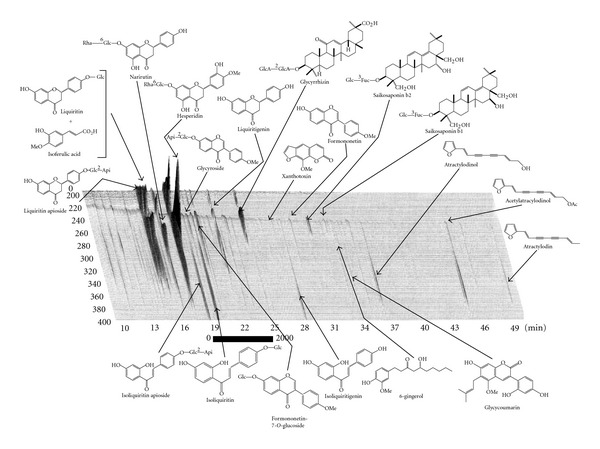
Chemical profile of TJ-41 analyzed by 3D HPLC. Each peak of TJ-41 in the HPLC profile was identified by comparison of the retention times and UV spectra of chemically defined standard compounds. HPLC condition was as follows: Column; Tosoh TSK GEL ODS-80Ts (4.6 × 250 mm). Carrier A: 0.05 M ammonium acetate (pH 3.6). Carrier B: acetonitrile. Gradient: 10%–100% carrier B linear in 60 min. Flow rate: 1.0 mL min^−1^. Injection volume: 30 micro liter. Detector: Shimadzu SPD-M10A VP.

Spray-dried extract preparation of Hochuekkito (Bu-Zhong-Yi-Qi-Tang in Chinese, TJ-41, batch no. 920041001PO) was kindly supplied from Tsumura & Co. (Tokyo, Japan). For the preparation of TJ-41, a mixture of Astragali Radix (4 g, roots of *Astragalus membranaceus* Bunge), Atractylodis lanceae Rhizoma (4 g, rhizomes of *Atractylodes lancea* DC.), Ginseng Radix (4 g, roots of *Panax ginseng* C.A. Meyer), Angelicae Radix (3 g, roots of *Angelica acutiloba* Kitagawa), Bupleuri Radix (2 g, roots of *Bupleurum falcatum* L.), Zizyphi Fructus (2 g, fruits of *Zizyphus jujuba* Miller var. *inermis* Rehder), Aurantii Bobilis Pericarpium (2 g, pericarps of ripe fruits of *Citrus unshu* Markovich), Glycyrrhizae Radix (1.5 g, roots of *Glycyrrhiza uralensis* Fisch *et* DC.), Cimicifugae Rhizoma (1 g, rhizomes of *Cimicifuga simplex* Wormskjord) and Zingiberis Rhizoma (0.5 g, rhizomes of *Zingiber officinale* Roscoe) was extracted with 285 mL of water by heating at 100°C for 1 h. The extracted solution was filtered and spray-dried to obtain dry extract powder (TJ-41, 5 g). Three-dimensional high performance liquid chromatography (HPLC) analysis was carried out to know chemical profile of TJ-41, and the spray-dried extract preparation of TJ-41 showing 3D-HPLC profile as in Figure 1 was used for the present study. Spray-dried extract preparation of Juzentaihoto (Shi-Quan-Da-Bu-Tang, TJ-48, batch no. 890048001PO) was also kindly supplied from Tsumura & Co. as described previously [10]. Arabino-3,6-galactan (ALR-5IIa-1-1), which has immunomodulating activity against Peyer's patch cells, was purified as a positive control from rhizomes of *Atractylodes lancea* according to the procedure of Yu et al. [17].

### 2.2. Cells

Establishment and characterization of murine normal colonic epithelial cell-line MCE301 were reported previously [18]. The MCE301 cells were cultured according to the procedure of Matsumoto et al. [16].

### 2.3. Animals

Specific pathogen-free female C3H/HeJ and BALB/c mice of 7 weeks old (young) and 6 months old (early aged) were purchased from Japan SLC (Shizuoka, Japan). The animals were housed in plastic gages in an air-conditioned room at 23 ± 2°C with a relative humidity of 55 ± 10% under a 12-h light-dark cycle, fed a standard laboratory diet and given water *ad libitum*. Animal experiments were approved by the Animal Research Committee of Kitasato University, and performed in accordance with Guidelines for Care and Use of Laboratory Animal at Kitasato University and the National Research Council Guide for the Care and Use of Laboratory Animals in Japan.

### 2.4. Analytical Methods

The carbohydrate, uronic acid and protein contents were determined by phenol—H_2_SO_4_ method [19], *m*-hydroxybiphenyl method [20] and Bradford method [21], respectively, using galactose, galacturonic acid and bovine immunoglobulin as the respective standards. Lignin contents were analyzed by acetyl bromide method [22]. Component sugars were analyzed as TMS methylglycoside derivatives [23] by GC on Hewlett-Packard Model 5890 series II gas chromatograph as described previously [16].

### 2.5. Fractionation of TJ-41

TJ-41 (100 g) was fractionated into five fractions; MeOH-soluble fraction (F-1, yield 73.3%), MeOH and water-insoluble fraction (F-2, yield 9.9%), dialyzable intermediate size ingredient fraction (F-3, yield 4.8%) and non-dialyzable intermediate size ingredient fraction (F-4, yield 0.2%), and macromolecular weight ingredient fraction (F-5, yield 7.7%) according to the method described previously [16].

### 2.6. Periodate Oxidation of F-2 and F-5 from TJ-41

Periodate oxidation was performed by the procedure as described previously [16].

### 2.7. Amylase Digestion of F-2 and F-5 from TJ-41

Amylase digestion was performed as described previously [16].

### 2.8. Chlorite Treatment of F-2 and F-5 from TJ-41

Solutions of F-2 and F-5 (1 g each) in 4% acetic acid (500 mL) were each added sodium chlorite (5 g), and incubated at 70°C for 40 min. The reaction mixtures were neutralized with 3 M NaOH, and then dialyzed against distilled water for 4 days. The non-dialyzable portions were concentrated by evaporation, and lyophilized to obtain chlorite-treated F-2 (yield 78.2%) and F-5 (yield 75.5%).

### 2.9. Purification of Polysaccharides and Lignin-Polysaccharide Complexes from F-5 

#### 2.9.1. Anion-Exchange Chromatography

A solution of F-5 (2.6 g) in 300 mL of distilled water was applied to a DEAE-Sepharose FF column (HCO_3_^−^), GE Healthcare Japan, 5.5 × 25 cm), and a neutral fraction (F-5I, 1.15 g, yield from F-5; 44.5%) was obtained by washing with distilled water. Absorbed fractions were eluted gradually with 50, 100, 200, 300, 400, 500, 600 mM and 1 M NH_4_HCO_3_ in a stepwise manner, and each eluate was dialyzed and lyophilized to obtain eight fractions; F-5IIa (50 mM NH_4_HCO_3_, 210 mg, yield from F-5; 8.1%), F-5IIb (100 mM NH_4_HCO_3_, 56 mg, 2.2%), F-5IIc (200 mM NH_4_HCO_3_, 249 mg, 9.6%), F-5IId (300 mM NH_4_HCO_3_, 97.3 mg, 3.8%), F-5IIe (400 mM NH_4_HCO_3_, 478 mg, 18.5%), F-5IIf (500 mM NH_4_HCO_3_, 38 mg, 1.5%), F-5IIg (600 mM NH_4_HCO_3_, 6 mg, 0.2%), and F-5IIh (1 M NH_4_HCO_3_, 19 mg, 0.7%).

#### 2.9.2. Gel Filtration

All acidic fractions, obtained from F-5 by anion-exchange chromatography except F-5IIg (because of too small amount available), were individually fractionated on a Sephacryl S-200 column (2.6 × 93 cm) with 0.2 M NaCl. All the resulting subfractions were dialyzed against distilled water, and lyophilized to give 17 different polysaccharide-containing fractions (PS1–PS17).

### 2.10. Measurement of In Vitro Immunomodulating Activity against Peyer's Patch Immunocompetent Cells

Immunomodulating activity was measured by enhanced production of bone marrow cells-proliferative cytokines from Peyer's patch cells of 7 weeks old female C3H/HeJ mice according to Hong et al. [24].

### 2.11. Measurement of G-CSF Production Enhancing Activity on MCE301 Cells

The procedure was performed in accordance with the method described previously [16].

### 2.12. Measurement of In Vivo Immunomodulating Activity of Fractions from TJ-41 on Upper Respiratory Mucosal Immune System

The procedures were carried out according to the method described previously [10]. Briefly, young or early aged BALB/c mice were immunized twice with a primary and secondary intranasal inoculations of 5 *µ*g of a split vaccine of influenza hemagglutinin (influenza A/NC/20 HA vaccine) (kind gift from Center for Biologicals of The Kitasato Institute) at Days 0 and 28. The mice were administered orally aqueous solutions of TJ-41 (100 mg kg^−1^ day^−1^) or each fraction (F-1 (73.3 mg kg^−1^ day^−1^), F-2 (9.9 mg kg^−1^ day^−1^), F-3 (4.8 mg kg^−1^ day^−1^), F-4 (0.2 mg kg^−1^ day^−1^) and F-5 (7.7 mg kg^−1^ day^−1^)) (doses were calculated by the yields of the respective fractions from TJ-41) from 7 days before the primary immunization (Day-7) to Day 42 once a day. The sera were prepared from blood samples of the mice at Day 42, and then nasal washes were taken from the posterior opening of the nasopharynx by injection with sterilized phosphate buffered saline containing 0.1% bovine serum albumin. The titers of influenza virus-specific IgG antibody (in sera) and IgA antibody (in nasal washes) were measured by ELISA according to the procedure as described previously [10].

### 2.13. Statistics

Data were expressed as mean ± SD, and differences between groups were analyzed by analysis of variance (ANOVA) followed by *post hoc* analyses using Scheffe's test. The probability (*P*) values < .05 were considered significant.

## 3. Results

### 3.1. Evaluation of Immunomodulating Activity of TJ-41 against Peyer's Patch Immunocompetent Cells

**Figure 2:**
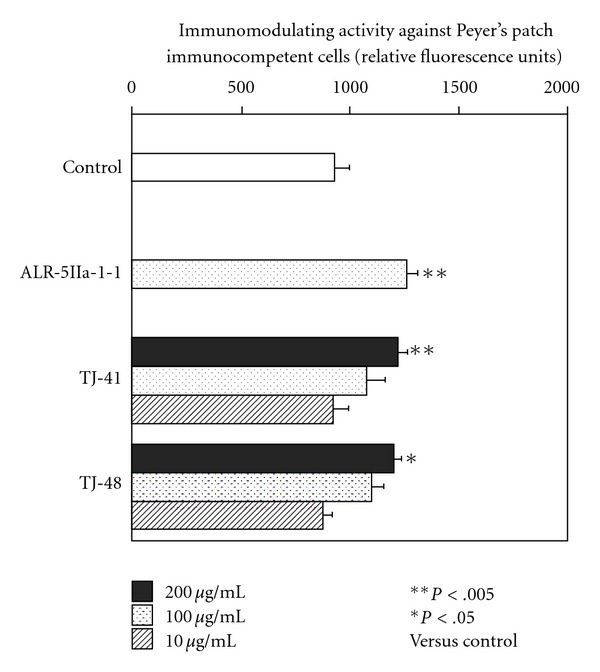
Comparison of immunomodulating activities of TJ-41 and TJ-48 against Peyer's patch immunocompetent cells. Peyer's patch cells from early aged C3H/HeJ mice (6-months old) were cultured in 96-well culture plate with 10, 100, or 200 *µ*g mL^−1^ of TJ-41 or TJ-48 for 6 days, and then bone marrow cells from young C3H/HeJ mice were further cultured with the culture supernatant of Peyer's patch cells. Relative amounts of hemopoietic growth factor in the culture supernatants of Peyer's patch cells were estimated as number of proliferated bone marrow cells, which were measured by Alamar Blue method. Data were expressed as mean ± SD (*n* = 4), and analyzed by ANOVA followed by *post hoc* analysis using Scheffe's test. ALR-5IIa-1-1 is a *β*-d-(1→3,6)-galactan-containing polysaccharide as positive control described previously [17].

In order to analyze whether TJ-41 is able to modulate directly functions of immunocompetent cells in Peyer's patches, after Peyer's patch cells from early aged (6 months old) C3H/HeJ mice were cultured with TJ-41 for 6 days, bone marrow cells from young (7 weeks old) C3H/HeJ mice were further cultured with the culture supernatant of Peyer's patch cells, and then relative amounts of hemopoietic growth factors in the culture supernatant produced from Peyer's patch cells were estimated as number of the proliferated bone marrow cells. The numbers of proliferated bone marrow cells significantly increased when Peyer's patch cells were cultured with TJ-41 (Figure 2). The degree of the enhancement was same as those when Peyer's patch cells were cultured with Juzentaihoto (TJ-48), which has been reported to have immunomodulating activity against Peyer's patch cells [24] (Figure 2).

### 3.2. Analysis of Active Ingredients for Immunomodulation of Peyer's Patch Immunocompetent Cells

**Figure 3:**
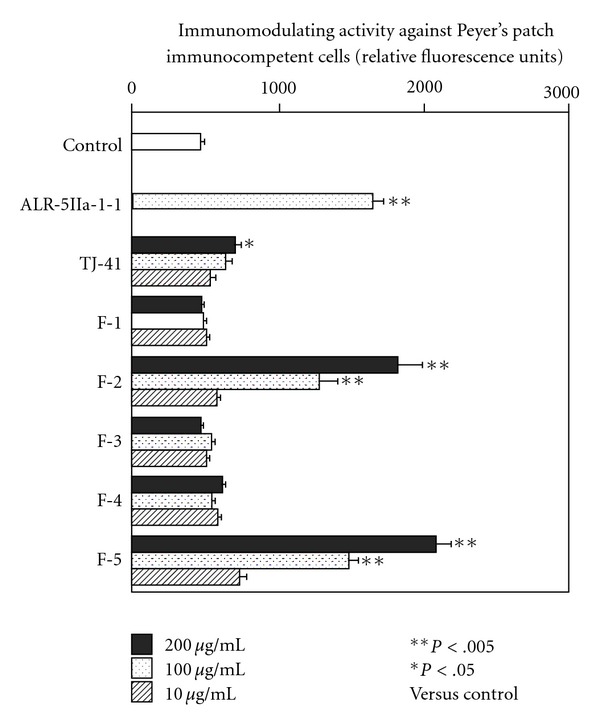
Immunomodulating activity against Peyer's patch immunocompetent cells of fractions prepared from TJ-41. Peyer's patch cells from early aged C3H/HeJ mice (6 months old) were cultured in 96-well culture plate with 10, 100 or 200 *µ*g mL^−1^ of TJ-41 or its fractions for 6 days, and then bone marrow cells from young C3H/HeJ mice were further cultured with the culture supernatant of Peyer's patch cells. Relative amounts of hemopoietic growth factor in the culture supernatants of Peyer's patch cells were estimated as number of proliferated bone marrow cells, which were measured by Alamar Blue method. Data were expressed as mean ± SD (*n* = 4), and analyzed by ANOVA followed by *post hoc* analysis using Scheffe's test. ALR-5IIa-1-1 is a *β*-d-(1→3,6)-galactan-containing polysaccharide as positive control described previously [17].

**Figure 4:**
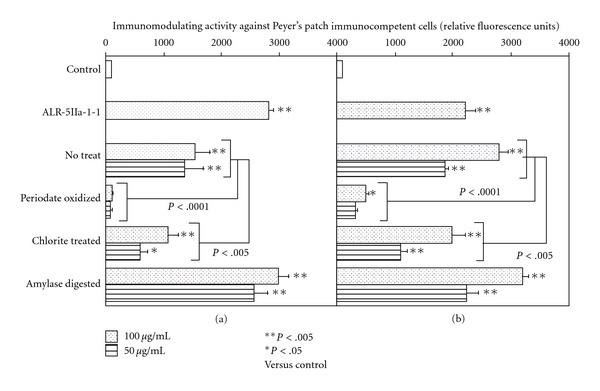
Effect of chemical and enzymic treatments of F-2 (a) and F-5 (b) on the immunomodulating activity against Peyer's patch immunocompetent cells of F-2 and F-5. Peyer's patch cells from early aged C3H/HeJ mice (6 months old) were cultured in 96-well culture plate with 50 or 100 *µ*g mL^−1^ of periodate, chlorite or amylase treated F-2 (a) or F-5 (b) for 6 days, and then bone marrow cells from young C3H/HeJ mice (7 weeks old) were further cultured with the culture supernatant of Peyer's patch cells. Relative amounts of hemopoietic growth factor in the culture supernatants of Peye's patch cells were estimated as number of proliferated bone marrow cells, which were measured by Alamar Blue method. Data were expressed as mean ± SD (*n* = 4), and analyzed by ANOVA followed by *post hoc* analysis using Scheffe's test. ALR-5IIa-1-1 is a *β*-d-(1→3,6)-galactan-containing polysaccharide as positive control described previously [17].

In order to elucidate which ingredient in TJ-41 contributes to expression of immunomodulating activity against Peyer's patch immunocompetent cells, TJ-41 was roughly fractionated by MeOH extraction, water extraction, EtOH precipitation and dialysis to obtain MeOH-soluble fraction (F-1), MeOH- and water-insoluble fraction (F-2), MeOH-insoluble and dialyzable fraction as dialyzable intermediate size ingredient fraction (F-3), EtOH-soluble and non-dialyzable fraction as non-dialyzable intermediate size ingredient fraction (F-4), and macromolecular weight ingredient fraction (F-5) [16]. When these five fractions were tested for immunomodulating activity against Peyer's patch cells, F-2 and F-5 showed statistically significant activity, but not the other fractions (Figure 3). Both F-2 and F-5 consisted mainly of carbohydrate (Table 1), and periodate oxidation, which is capable to degrade carbohydrate moieties, significantly decreased activities of both F-2 and F-5 (Figure 4). Because F-2 and F-5 mainly comprised glucose as a component sugar (Table 1), suggesting the presence of starch-like polysaccharide in these fractions, both fractions were also digested with *α*- and *β*-amylases. Amylase digestion did not reduce activities of both F-2 and F-5 (Figure 4). Component sugar analyses indicated that the amylase-digested F-2 and F-5 reduced contents of glucose significantly, and consisted mainly of galacturonic acid in addition to notable proportions of arabinose and galactose (Table 1). When F-2 and F-5 each were treated with sodium chlorite in order to destroy lignin molecules, the reaction products reduced the activity, but the considerable activity still remained for both F-2 and F-5 (Figure 4). These results suggested that F-2 and F-5 contained polysaccharides and/or lignin-polysaccharide complexes as active ingredients, and it was assumed that these active macromolecules in TJ-41 were separately fractionated into F-2 (water-insoluble fraction) and F-5 (water-soluble fraction) depending on their solubility in water.

**Figure 5:**
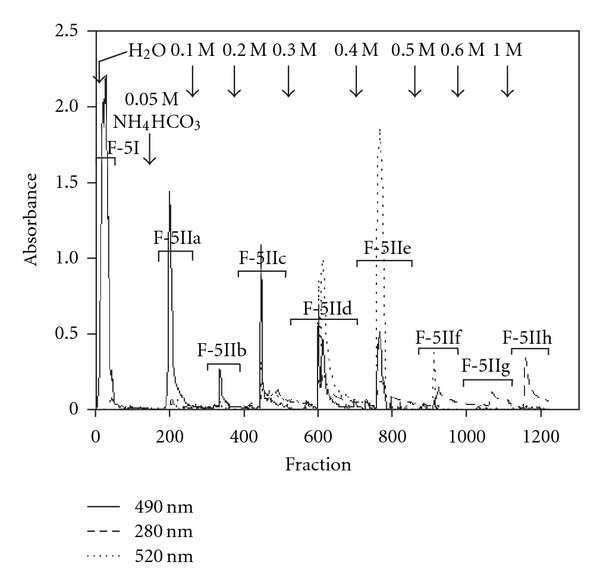
Elution pattern in anion-exchange chromatography of F-5 on DEAE-Sepharose. F-5 was applied to a DEAE-Sepharose FF column (HCO_3_^−^form), and the neutral fraction (F-5I) was eluted with water. Eight absorbed fractions were gradually eluted from the column with increased concentrations of HCO_3_^−^ solution as shown in this figure by stepwise manner. The eluted respective fractions were monitored for carbohydrate by phenol-sulfuric acid method (490 nm), uronic acid by *m*-hydroxybiphenyl method (520 nm) and phenolics or proteins by ultraviolet absorption at 280 nm.

**Figure 6:**
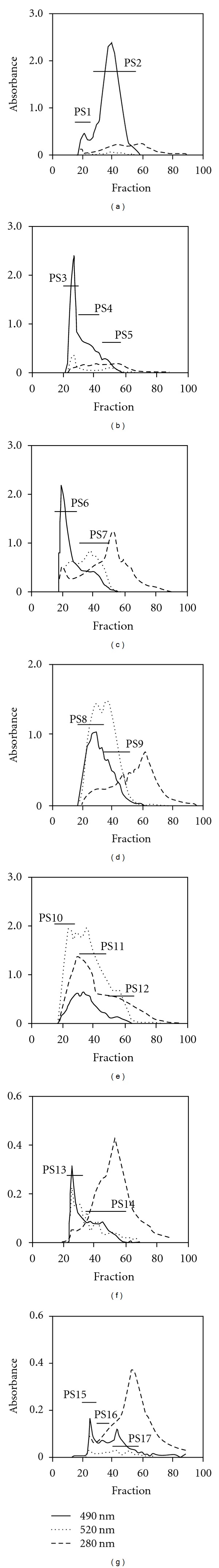
Elution patterns in size exclusion chromatography of fractions obtained from Figure 5 on Sephacryl S-200. F-5IIa (a), F-5IIb (b), F-5IIc (c), F-5IId (d), F-5IIe (e), F-5IIf (f) and F-5IIh (g) from Figure 5 were applied to a Sephacryl S-200 column equilibrated with 0.2 M NaCl, and eluted with the same solution. The eluted respective fractions were monitored for carbohydrate by phenol-sulfuric acid method (490 nm), uronic acid by *m*-hydroxybiphenyl method (520 nm) and phenolics or proteins by ultraviolet absorption at 280 nm.

**Figure 7:**
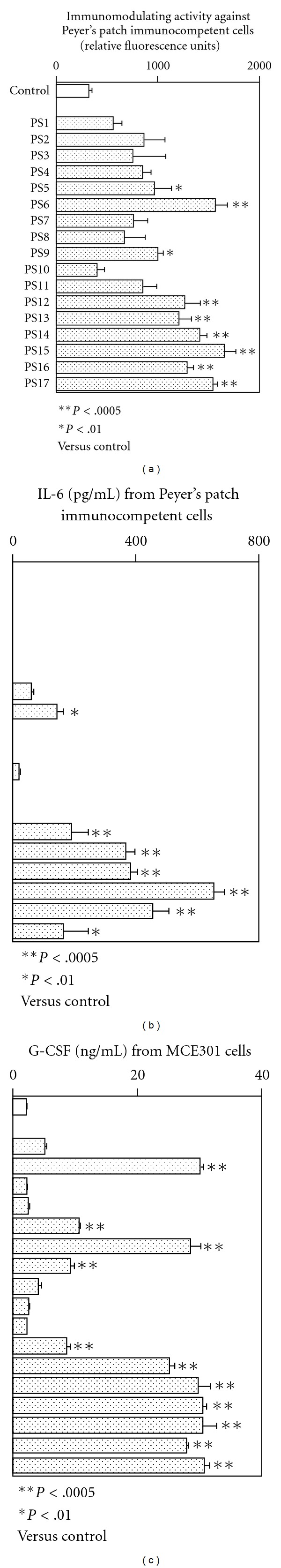
Immunomodulating activities against Peyer's patch immunocompetent cells and intestinal epithelial cells of polysaccharide-containing subfractions obtained from macromolecular weight ingredient fraction (F-5) of TJ-41. (a) Immunomodulating activities against Peyer's patch immunocompetent cells of the subfractions obtained from Figure 6. Peyer's patch cells from early aged C3H/HeJ mice (6 months old) were cultured in 96-well culture plate with 100 *µ*g mL^−1^ of the subfractions for 6 days, and then bone marrow cells from young C3H/HeJ mice (7 weeks old) were further cultured with the culture supernatant of Peyer's patch cells. Relative amounts of hemopoietic growth factor in the culture supernatants of Peyer's patch cells were estimated as number of proliferated bone marrow cells, which were measured by Alamar Blue method. (b) IL-6 production enhancing activity on Peyer's patch immunocompetent cells of the subfractions from Figure 6. Peyer's patch cells from early aged C3H/HeJ mice (6 months old) were cultured in 96-well culture plate with 100 *µ*g mL^−1^ of the subfractions for 6 days, and IL-6 content in the culture supernatant was measured using an ELISA. (c) G-CSF production enhancing activity on intestinal epithelial cells of the subfractions from Figure 6. Murine colonic epithelial cell line, MCE301 cells were cultured for 48 h in 96-well culture plate with 0.5 *µ*g mL^−1^ of the subfractions, and G-CSF content in the culture supernatant was measured using an ELISA. All data were expressed as mean ± SD (*n* = 4), and analyzed by ANOVA followed by *post hoc* analysis using Scheffe's test.

F-5 was further fractionated by anion-exchange chromatography on a DEAE-Sepharose FF column (HCO_3_^−^) and one neutral (F-5I) and 8 acidic fractions (F-5IIa–F-5IIh) were obtained (Figure 5). All acidic fractions consisted of arabinose, galactose, glucose and/or galacturonic acid whereas the neutral fraction (F-5I) mainly comprised glucose (data not shown). Although F-5I did not show immunomodulating activity against Peyer's patch cells, all acidic fractions had significant activity (data not shown). Therefore, the acidic fractions were further fractionated by gel filtration except F-5IIg because of too small amount available. By gel filtration on a Sephacryl S-200 column, F-5IIa, IIc, IId, and IIf each gave two subfractions (PS1 and PS2 (from F5-IIa), PS6 and PS7 (from F-5IIc), PS8 and PS9 (from F-5IId), and PS13 and PS14 (from F-5IIf)), which were rich in carbohydrate (Figure 6). Whereas, F-5IIb, IIe and IIh gave three carbohydrate-rich subfractions each (PS3, PS4, and PS5 (from F-5IIb), PS10, PS11, and PS12 (from F-5IIe), and PS15, PS16, and PS17 (from F-5IIh)). Analyses of chemical properties indicated that PS1, PS2, PS3, PS4, PS5, PS11, PS12, and PS13 consisted mainly (over 80%) of carbohydrate (total of carbohydrate and uronic acid contents in Table 2), whereas PS7, PS8, PS9, PS10, and PS14 comprised about 60–70% of carbohydrate (Table 2). Meanwhile, PS6, PS15, PS16, and PS17 contained <60% of carbohydrate. All subfractions contained different amount of lignin, especially, PS9, PS12, PS14, PS16 and PS17 were composed of over 20% of lignin (Table 2). When these subfractions were analyzed immunomodulating activity against Peyer's patch cells, PS5, PS6, PS9, PS12, PS13, PS14, PS15, PS16 and PS17 showed the significant activity, and the other subfractions seemed to have weak activity except PS10 although statistical significances were not be observed (Figure 7(a)). Because TJ-41 up-regulated mRNA expression of interleukin-6 (IL-6) on Peyer's patch immunocompetent cells (data not shown), the effect of these subfractions on production of IL-6 from Peyer's patch cells were also evaluated. As shown in Figure 7(b), PS6, PS12, PS13, PS14, PS15, PS16 and PS17 significantly enhanced IL-6 production from Peyer's patch cells.

### 3.3. Evaluation of Modulating Activity of Polysaccharide-Containing Macromolecules from TJ-41 on G-CSF Production of Intestinal Epithelial Cells

**Figure 8:**
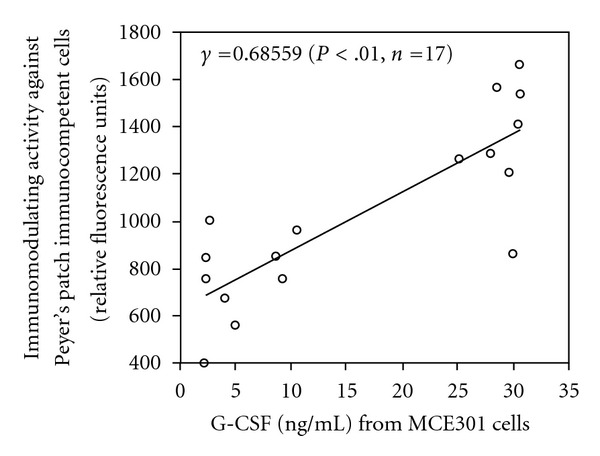
Positive significant correlation between degrees of immunomodulating activity against Peyer's patch immunocompetent cells and G-CSF production enhancing activity on intestinal epithelial cells of polysaccharide-containing subfractions in TJ-41. Two-dimensional scatter plot was made from the data of immunomodulating activity against Peyer's patch immunocompetent cells and G-CSF production enhancing activity on MCE301 cells of 17 subfractions obtained from Figure 6. Regression line was calculated by least squares method, and estimated Pearson's correlation coefficient (*γ*).

Previous study has indicated that TJ-41 enhances *de novo* G-CSF synthesis on the murine colonic epithelial cell line, MCE301, and that only macromolecular weight ingredient fraction (F-5) of TJ-41 has G-CSF production enhancing activity on MCE301 [16]. Therefore, PS1–PS17 obtained from F-5 in the present study were also analyzed effects on G-CSF production of MCE301. As shown in Figure 7(c), PS2, PS5, PS6, PS7, PS11 and PS12–PS17 significantly enhanced G-CSF production at a concentration of 0.5 *µ*g mL^−1^. Simple linear regression analysis was performed between degrees of immunomodulating activity against Peyer's patch cells and G-CSF production enhancing activity on MCE301 cells of these subfractions. The statistically significant positive correlation (*γ* = 0.68559, *P* < .01, *n* = 17) between both activities was observed (Figure 8), and suggesting that these subfractions modulate functions of not only Peyer's patch immunocompetent cells but also intestinal epithelial cells.

### 3.4. Contribution of Polysaccharide-Containing Macromolecules for Immunomodulating Activity of TJ-41 on Upper Respiratory Mucosal Immune System

**Figure 9:**
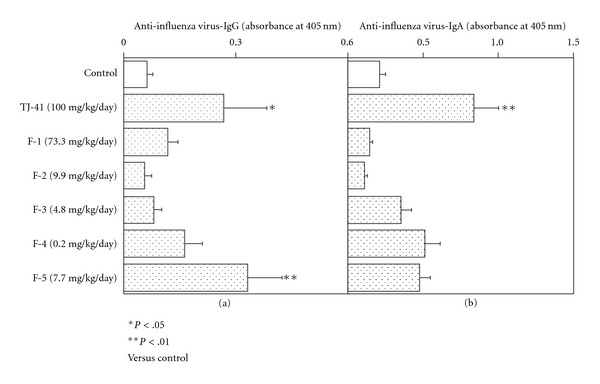
Effect of oral administration of TJ-41 or its fractions on antibody responses in systemic immune system and upper respiratory mucosal immune system against nasal immunization. (a) Enhancing activity on influenza virus-specific IgG antibody response in systemic immune system against nasal immunization in young mice. Young BALB/c mice (7 weeks old) were intranasally immunized twice with influenza vaccine, and TJ-41 (100 mg kg^−1^ day^−1^) or its subfractions (each daily dose was calculated to be equivalent to 100 mg kg^−1^ day^−1^ of TJ-41 from the yield of each fraction) were orally administered to the immunized mice. Sera were taken from the mice, and antiinfluenza virus-specific IgG antibody titer in the sera was measured by ELISA. (b) Enhancing activity on influenza virus-specific IgA antibody response in nasal cavity against nasal immunization in early aged mice. Early aged BALB/c mice (6 months old) were intranasally immunized twice with influenza vaccine, and TJ-41 (100 mg kg^−1^ day^−1^) or its fractions (each daily dose was calculated to be equivalent to 100 mg kg^−1^ day^−1^ of TJ-41 from the yield of each fraction) were orally administered to the immunized mice. Nasal washes were taken from the mice, and anti-influenza virus-specific IgA antibody titer in the washes was measured by ELISA. All data were expressed as mean ± SD (*n* = 8), and analyzed by ANOVA followed by *post hoc* analysis using Scheffe's test.

It has been found that oral administration of TJ-41 stimulates antigen-specific antibody production on only systemic immune system against nasally inoculated antigen to the young mice (7 weeks old) [10], whereas TJ-41 enhances antibody production on upper respiratory tract of early aged mice (6 months old) [10]. In order to evaluate whether polysaccharide-containing macromolecules in TJ-41 contribute to expression of enhancing activity on upper respiratory mucosal immune system of TJ-41, each of F-1–F-5 obtained from TJ-41, was orally administered at doses related to their yields from TJ-41 to young or early aged BALB/c mice, which were nasally immunized twice with influenza vaccine, and titers of influenza virus specific IgG or IgA antibody were measured for sera or nasal washes from the mice, respectively. Oral administrations of F-1, F-2, F-3, and F-4 failed to increase influenza virus specific IgG antibody titer in sera of young mice, whereas F-5 significantly enhanced the antibody titer as same as TJ-41 (Figure 9(a)). However, when these fractions each were administered to early aged mice, all fractions did not enhance influenza virus specific IgA antibody titer in nasal cavity even though TJ-41 could (Figure 9(b)).

## 4. Discussion

In previous study TJ-41 has been found to potentiate antibody response in upper respiratory mucosal immune system *in vivo* but not another Kampo formula, TJ-48, and it has been suggested that TJ-41 express this stimulating activity through intestinal immune system [10]. The present study indicated that TJ-41 directly modulated immunocompetent cells in Peyer's patches to enhance production of bone marrow cell-proliferative growth factors *in vitro*. However, significant difference could not be observed between TJ-41 and TJ-48. When mRNA expressions of cytokines were analyzed by PCR using cells which were cultured with either TJ-41 or TJ-48 for 6 days, enhanced mRNA expressions of IL-2, IL-5, IL-6, IL-10 and TGF-*β * were observed in cultured Peyer's patch cells with TJ-41 compared with those in cells cultured without the formula (Kiyohara & Nonaka, unpublished data). Meanwhile, TJ-48 significantly enhanced mRNA expression of only IL-2 (Kiyohara & Nonaka, unpublished data). These results postulated that TJ-41 might modulate cytokine productions in different manner from TJ-48. The difference may bring to one of reasons why TJ-41 can potentiate upper respiratory mucosal immune system. It is necessary to analyze how TJ-41 and TJ-48 differently modulate cytokine production on immunocompetent cells in Peyer's patches *in vivo* in future study.

Numerous ingredients are considered to be present in TJ-41 because this multiherbal formula consists of 10 kinds of component herbs. The present study indicated that macromolecular weight ingredients in F-5 were responsible active ingredients for immunomodulation of Peyer's patch immunocompetent cells. All macromolecular weight ingredients in TJ-41 comprised various proportions of polysaccharides and lignins. Polysaccharides and lignins are primary metabolites, and lignins are generally conjugated with poly- and oligosaccharides to form lignin-polysaccharide complexes in plant cell walls [25, 26]. The present study suggests that both structural moieties are required for expression of modulating activity on Peyer's patch cells because the activity was significantly reduced by both periodate oxidation and chlorite treatment. However, no correlation was observed between lignin contents and degrees of the immunomodulating activity on Peyer's patch cells of 17 subfractions, and it is assumed that detailed structural parts of both lignin and polysaccharide moieties may participate to expression of the immunomodulating activity. We have already found a immunomodulating polysaccharide (ALR-5IIa-1-1 used as a positive control in the present study) against Peyer's patch cells from *Atractylodes lancea* as one of component herbs of TJ-41 [17], and this polysaccharide was shown to express the activity through *β*-d-(1→3,6)-galactan moiety as the active carbohydrate sequence [27]. Although ALR-5IIa-1-1 clearly decreased the activity by an enzymatic degradation of *β*-d-(1→3,6)-galactan moiety [27], the immunomodulating activity against Peyer's patch cells of some of the fractions obtained from F-5 of TJ-41 could not be reduced by the same enzymatic degradation for *β*-d-(1→3,6)-galactan moiety (Kiyohara & Sekiya, unpublished data). These results assumed that the active polysaccharides and lignin-polysaccharide complexes in TJ-41 have different responsible structures from ALR-5IIa-1-1 for expression of the activity.

Intestinal epithelial cells sit at interface between lumen and lymphocyte-rich lamina propria. The epithelial cells function as effector/regulator cells of a host's immune response to foreign substances including various pathogens and food antigens [13, 14]. Matsumoto et al. have suggested that certain ingredients in F-5 of TJ-41 contribute to express G-CSF production enhancing activity on intestinal epithelial cells [16]. A question arises whether Peyer's patch cells-modulating polysaccharides and/or lignin-polysaccharide complexes, isolated from TJ-41 in the present study, also have enhancing activity on G-CSF production of intestinal epithelial cells. Correlation analysis strongly suggests that immunomodulating polysaccharides and/or lignin-polysaccharide complexes against Peyer's patch cells in TJ-41 also interact with intestinal epithelial cells to affect on their immunological functions. This proposes that polysaccharides and/or lignin–polysaccharide complexes play as dual active ingredients for immunomodulation of immunocompetent cells in Peyer's patches and intestinal epithelial cells. It has been reported that carbohydrate moiety of polysaccharide and/or lignin-polysaccharide complexes in F-5 participate to enhancing activity of G-CSF production of intestinal epithelial cells [16]. However, structural features of the responsible carbohydrate moiety in the subfractions from F-5 also could not be analyzed in the present study, and further study needs to be carried out for clarification of responsible structures for immunomodulation of intestinal epithelial cells and immunocompetent cells in Peyer's patches.

Another question arises whether only macromolecular weight ingredients in TJ-41 behave as responsible molecules for expression of stimulating activity on upper respiratory mucosal immune system of TJ-41. The present study indicated that oral administration of only polysaccharides and/or lignin-polysaccharide complexes in F-5 potentiates antibody response against nasal inoculated antigen in systemic immune system. Antibody response against nasal immunization in systemic immune system is known to be induced as a result of migration of antigen-specific lymphocytes from NALT to spleen and peripheral lymph nodes [7–9]. Parts of naive and activated lymphocytes in Peyer's patches are generally considered to migrate into spleen and peripheral lymph nodes to interact with lymphocytes which are recruited from NALT [11]. Therefore it is hypothesized that antigen-specific antibody productions in spleen and peripheral lymph nodes may be enhanced by interaction between lymphocytes from NALT and Peyer's patch lymphocytes which were functionally modulated by the actions of polysaccharides/lignin-polysaccharide complexes of TJ-41. Since the possibility that these macromolecular weight ingredients directly enhance systemic immune system after their absorption from digestive system is not ruled out, detailed mechanism for this stimulatory effect is necessary to investigate by further study.

**Figure 10:**
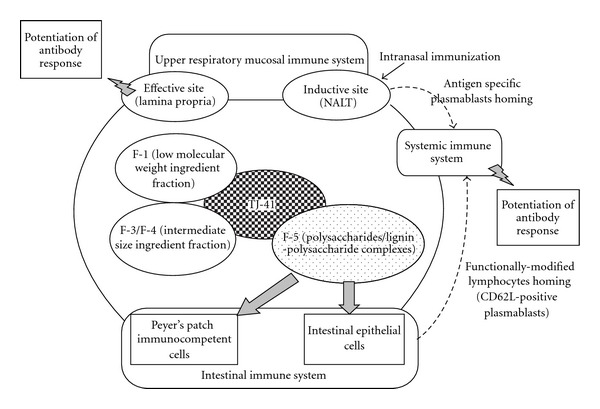
Hypothetical diagram on responsible ingredients in Hochuekkito (TJ-41) for expression of stimulating activity on antibody response against intranasal immunization.

The present study indicated that not only macromolecular weight ingredients (F-5) but also other ingredients (F-1, F-2, F-3, and F-4) of TJ-41 could not stimulate antibody response in upper respiratory tract against nasal immunization unlike TJ-41. This result strongly postulates that stimulation of upper respiratory mucosal immune system by the action of TJ-41 may be expressed by contributions of numerous ingredients in the fractions, F-1 to F-5 of TJ-41. It is generally known that lymphocytes are recruited to upper respiratory mucosal immune system from both hemopoietic system and Peyer's patches to control the immune system [7–9]. Process of lymphocyte homing from Peyer's patches to upper respiratory mucosal immune system is regulated by many molecules: (i) adhesive molecules like CD62L (L-selectin) and integrin *a*4*β*1, and chemokine receptors (CCR7 and CCR10) on lymphocytes, (ii) ligands for adhesion molecules like 6-sulfosialy Lewis^X^-expressing glycoprotein (PNAd) and VCAM-1, and chemokine like CCL28 on high endothelial venule (HEV) in upper respiratory tract [7–9]. It is difficult to consider that macromolecular weight ingredients of TJ-41 modulate expression of all above homing-related molecules. Therefore, it is hypothesized that TJ-41 may contain some other active ingredients, which can modulate expression of these homing-related molecules in addition to polysaccharides/lignin-polysaccharides, which modulate functions of immunocompetent cells in Peyer's patches and intestinal epithelial cells (Figure 10). Further study concerning with clarification of modulating ingredients on expression of homing-related molecules in TJ-41 may lead us to understand detailed mechanism for immunomodulation of upper respiratory mucosal immune system by the action of TJ-41.

## Figures and Tables

**Table 1:** Chemical properties of F-2 and F-5 and their chemical and enzymatic treated products.

	F-2	F-2 Amylase digested	F-2 Periodate oxidized	F-2 Chlorite treated	F-5	F-5 Amylase digested	F-5 Periodate oxidized	F-5 Chlorite treated
Carbohydrate content (%)	65.2	36.5	19.2	101.8	105.3	62.7	37.9	102.8
Uronic acid content (%)	7.3	14.4	3.6	8.2	19.2	29.9	4.2	20.3
Protein content (%)	4.1	10.8	26.4	11.6	7.5	15.6	13.4	14.0
Component sugar (mol.%)								
Arabinose	3.4	9.9	n.d.^a^	2.2	10.8	16.3	11.6	6.8
Rhamnose	0.5	1.4	n.d.	0.3	2.1	1.8	3.0	0.9
Fucose	0.1	0.6	n.d.	0.1	0.3	0.3	n.d.	0.2
Xylose	1.0	1.8	n.d.	0.8	0.9	1.8	2.5	1.3
Glucuronic acid	0.6	trace	trace	trace	0.5	trace	trace	trace
Galacturonic acid	5.5	48.0	70.4	10.7	18.0	63.0	46.3	30.4
Mannose	trace	5.0	3.2	1.1	trace	1.7	race	trace
Galactose	2.1	12.6	13.1	4.8	6.2	9.4	29.9	4.3
Glucose	86.9	20.9	13.4	80.0	61.4	5.7	6.6	56.1

^a^Not detected.

**Table 2:** Chemical properties of subfractions obtained from macromolecular weight fraction (F-5) of TJ-41.

	Carbohydrate content	Uronic acid content	Protein content	Lignin content	Component sugar (mol. %)								
	(%)	(%)	(%)^a^	(%)	Arabinose	Rhamnose	Fucose	Xylose	Glucuronic	Galacturonic	Mannose	Galactose	Glucose
									acid	acid			
PS1	72.8	13.3	7.5	9.1	41.5	1.1	0.3	5.7	0.8	n.d.	2.0	43.0	5.6
PS2	115.9	14.5	11.5	10.3	7.9	1.3	0.3	7.1	0.5	3.8	11.9	8.5	56.8
PS3	89.0	19.3	7.5	11.3	41.1	2.5	0.2	5.7	2.1	0.5	1.5	45.0	1.4
PS4	73.8	10.7	10.7	13.0	34.5	4.8	0.4	11.1	2.3	1.9	6.8	32.8	5.2
PS5	83.2	16.2	7.8	6.2	40.8	6.1	0.3	5.1	4.9	8.4	2.6	15.0	16.7
PS6	24.9	21.4	18.7	14.9	16.4	7.4	1.1	2.1	3.0	41.1	6.9	6.5	15.5
PS7	37.9	24.3	7.3	7.4	26.8	7.9	0.5	2.8	3.5	37.0	n.d.	6.9	14.7
PS8	28.5	49.6	10.5	14.6	14.1	5.4	0.8	2.2	1.1	66.6	n.d.	2.8	7.0
PS9	6.7	60.6	11.8	21.1	4.7	1.8	0.5	1.4	4.9	73.4	n.d.	2.4	10.9
PS10	38.7	28.4	14.1	9.1	14.7	4.0	0.7	2.1	7.9	61.2	n.d.	3.2	6.3
PS11	29.7	78.6	15.6	10.9	7.8	2.3	0.7	1.3	3.0	74.7	n.d.	3.4	6.8
PS12	26.7	62.7	17.3	20.1	9.1	2.8	0.8	1.5	6.2	62.3	3.7	3.3	10.3
PS13	34.3	67.9	16.3	16.4	16.6	4.8	0.7	2.3	3.8	41.2	10.8	10.2	9.6
PS14	17.9	43.0	20.2	32.1	10.0	3.3	0.9	2.3	3.1	44.3	15.8	6.1	14.2
PS15	27.1	28.8	22.5	16.4	18.9	4.2	0.4	3.5	4.3	18.6	15.2	19.6	15.2
PS16	22.7	14.9	25.8	21.1	18.0	3.7	0.5	5.7	7.6	19.3	15.2	9.2	20.3
PS17	11.7	20.5	17.6	36.2	13.7	6.0	0.7	4.6	4.2	23.7	19.2	5.6	22.4

^a^Bradford method has been reported to cross react with lignin [25]. Therefore the values of protein contents did not indicate exact contents of protein in the subfractions.

n.d., Not detected.
